# The Potential Therapeutic Application of Peptides and Peptidomimetics in Cardiovascular Disease

**DOI:** 10.3389/fphar.2016.00526

**Published:** 2017-01-06

**Authors:** Carlota Recio, Francesco Maione, Asif J. Iqbal, Nicola Mascolo, Vincenzo De Feo

**Affiliations:** ^1^Sir William Dunn School of Pathology, University of OxfordOxford, UK; ^2^Department of Pharmacy, University of Naples Federico IINaples, Italy; ^3^Department of Pharmacy, University of SalernoSalerno, Italy

**Keywords:** cardiovascular disease, cardiovascular system, inflammation, peptides, peptidomimetics

## Abstract

Cardiovascular disease (CVD) remains a leading cause of mortality and morbidity worldwide. Numerous therapies are currently under investigation to improve pathological cardiovascular complications, but yet, there have been very few new medications approved for intervention/treatment. Therefore, new approaches to treat CVD are urgently required. Attempts to prevent vascular complications usually involve amelioration of contributing risk factors and underlying processes such as inflammation, obesity, hyperglycaemia, or hypercholesterolemia. Historically, the development of peptides as therapeutic agents has been avoided by the Pharmaceutical industry due to their low stability, size, rate of degradation, and poor delivery. However, more recently, resurgence has taken place in developing peptides and their mimetics for therapeutic intervention. As a result, increased attention has been placed upon using peptides that mimic the function of mediators involved in pathologic processes during vascular damage. This review will provide an overview on novel targets and experimental therapeutic approaches based on peptidomimetics for modulation in CVD. We aim to specifically examine apolipoprotein A-I (apoA-I) and apoE mimetic peptides and their role in cholesterol transport during atherosclerosis, suppressors of cytokine signaling (SOCS)1-derived peptides and annexin-A1 as potent inhibitors of inflammation, incretin mimetics and their function in glucose-insulin tolerance, among others. With improvements in technology and synthesis platforms the future looks promising for the development of novel peptides and mimetics for therapeutic use. However, within the area of CVD much more work is required to identify and improve our understanding of peptide structure, interaction, and function in order to select the best targets to take forward for treatment.

## Introduction

Cardiovascular disease remains a leading cause of mortality and morbidity worldwide. In developed countries, risk factors such as hypertension, hyperglycemia, and hypercholesterolemia are accepted as having a key role in driving CVD (Leening et al., 2016). Researchers and clinicians have spent significant time and effort investigating the role of these risk factors in the development and progression of CVD, yet there have been a limited number of new medications approved for CVD-related intervention and/or treatment. Therefore, new approaches to treat CVD are needed. Attempts to prevent vascular complications usually involve amelioration of contributing risk factors and underlying processes such as inflammation, obesity, hyperglycaemia, or hypercholesterolemia (Navickas et al., 2016; Pirlamarla and Bond, 2016).

Targeting lipids has been the major strategy used in treating CVD to date. Hypercholesterolemia plays a key role in peripheral coronary artery disease progression, mainly atherosclerosis. High concentration of low density-lipoprotein (LDL) particles in plasma drives cholesterol accumulation in arteries setting up the initial stage of atheroma plaque formation (Libby et al., 2011; Manduteanu and Simionescu, 2012). Excessive lipid accumulation in the arterial intima induces a significant inflammatory response resulting in increased pro-inflammatory cytokines, adhesion molecules, and chemokine expression, which leads to endothelial dysfunction and leukocyte infiltration (Manduteanu and Simionescu, 2012; Schett et al., 2013; LeBert and Huttenlocher, 2014). Further influx (mainly macrophages, T cells, and vascular smooth muscle cells) of cells into the lesion area triggers plaque hardening and growth. Finally, vessel diameter decreases and, if the plaque is unstable, it can cause significant clinical consequences such MI or stroke (Fuster et al., 2005).

The current first line drugs used in CVD treatment to date are ACEIs, ARBs, anticoagulants, cholesterol-lowering drugs (statins), beta-blockers, and some anti-inflammatory medicines (NSAID, glucocorticoids). The majority of these drugs have shown efficacy but many are also associated with a wide range of side-effects and are therefore inadequate to use in long-term treatment regimens (Nathan, 2002; Lawrence et al., 2002; Costopoulos et al., 2013; Cheng et al., 2014; Pellicori and Costanzo, 2015; Stein and Raal, 2015).

This review will explore novel targets and experimental therapeutic approaches based on peptidomimetics for modulation in CVD including atherosclerosis, vascular diabetic complications and MI, among others.

## Peptides as Therapeutics

Therapeutic peptides are described as naturally occurring short amino acid monomer chains, shorter than 100 amino acids, and they act by binding to specific cell surface receptors, where they trigger intracellular pathways (Vlieghe et al., 2010). They have been shown to possess desirable pharmacological profiles and their specificity has been seen to translate into outstanding safety, tolerability, and efficacy profiles in humans, in stark contrast to traditional small molecules (Vlieghe et al., 2010; Goodwin et al., 2012).

The idea of using peptides as therapeutic agents has been historically ignored by pharmaceutical companies due to several limitations including size, which makes them very susceptible to degradation by peptidases, the lack of effective methods for delivery, poor transport properties through biologic membranes, low oral bioavailability, rapid excretion, and poor target specificity resulting from the flexible nature of peptides (**Table 1**) (Vagner et al., 2008). More recently, however, in light of advances in processing technologies, there has been a renewed interest in peptides and peptidomimetics as potential therapeutic agents. This is partly due to numerous improvements made to stability, transport, affinity profiles, and oral availability (Goodwin et al., 2012; Fosgerau and Hoffmann, 2015). Furthermore, the introduction of alternative delivery methods by new adjuvant and carrier systems have been developed, and the advance of proteomics identifying innumerable PPI targets, has increased the interest in peptides and their mimetics as potential therapeutic drugs (Liskamp et al., 2011; Akram et al., 2014).

**Table 1:** Advantages and disadvantages of peptides as therapeutics.

Advantages	Disadvantages
Broad range of targets	Limited oral bioavailability
Low toxicity	Elevated production costs
High chemical and biological diversify	Short half-life and rapid clearance
High potency and selectivity	Low metabolic stability
Good efficacy, safety, and tolerability	Poor membrane permeability
Low accumulation in tissues	Tendency for aggregation
Standard synthetic protocols	They can contain immunogenic sequences

To date, more than 7000 naturally occurring peptides have been described (Fosgerau and Hoffmann, 2015). The first chemical synthesis of a therapeutic peptide was that of oxytocin in 1953. Recombinant synthesis of proteins was introduced in 1974, and recombinant human insulin, the first approved therapeutic peptide to be manufactured by recombinant fermentation, was introduced in 1982 (Puttagunta and Toth, 1998). Although being used for the last five decades, it still enjoys the fame of being the most generally prescribed peptide worldwide (McGill et al., 2016). At present, there are more than 60 peptide-based drug products that have reached approval and nearly 140 in clinical trials (Lax and Meenan, 2012; Uhlig et al., 2014).

To address these key technical hurdles to use peptides as medicines, a number of modifications strategies (thanks to robust peptide-chemistry approaches developed in recent years) have been widely adopted. Several bioactive peptides have proven to be highly functional with many serving as potent agonists and antagonists against numerous receptors implicated in disease progression (Kaspar and Reichert, 2013; Peptide Therapeutics Market, 2015).

Transformation of peptides to peptidomimetics is one intriguing mechanism to use peptide sequences as potential therapeutic agents. Peptides can be adapted to stable mimics that expose similar effects to their peptide analog but show increased consistency in structure, more target specificity, increased stability to proteolytic digestion and greater cell membrane permeability. Therefore, peptides have been chemically altered to include unnatural amino acid substitutions, backbone amide bond modifications, or rigid scaffolds or the addition of hydrophobic residues (Gentilucci et al., 2006; Vagner et al., 2008; Vlieghe et al., 2010).

Among the major drawbacks faced by the use of peptides as drugs is their delivery. To date, injections remain the most common route of administration. However, oral delivery would be preferable because of its high level of patient compliance which increases the therapeutic value of a drug. So the challenge remains to improve the oral bioavailability from less than 1% to at least 30–50%. Recently, development of orally and nasal active preparations have been proposed as a result of encapsulation of peptides in nanoparticles (e.g., liposomes, synthetic polymers, or fullerenes) which shields the drug from protease digestion until required, therefore increasing stability (Mason, 2010; Bruno et al., 2013). Other strategies under investigation to overcome peptide barriers include use of protease inhibitors, absorption enhancers, or conjugated molecules in combination with the peptide structure, such as antibodies to improve targeting, carbohydrates to increase solubility, or lipids to enhance peptide permeability (Shaji and Patole, 2008; Bruno et al., 2013; Di, 2015)

Equally, synthesis costs are a big issue. The synthesis of peptides relies heavily on expensive coupling reagents, resins and protected amino acids, so cheaper methods for their synthesis and purification are required (Mason, 2010). In line with this, chemical methods such as click chemistry and peptide synthesis reactors that can handle large amounts of reaction material for solid-phase peptide synthesis can substantially lower costs as well as improve the chemistry (Sohma et al., 2004; Fabbrizzi et al., 2014).

Another major breakthrough has been the variety drive in technology platforms to study PPIs. With more information related to 3D structure of protein complexes and PPIs and their importance in human diseases, peptide- and peptidomimetic-based therapeutic agents have become a major area of drug design, competing with natural products, synthetic small molecules, and antibody-based therapies (Gao et al., 2015).

Considering all the upgrades in peptide systems, and the rapid developments in proteomics, bioinformatics, and peptide libraries, it is expected that by 2020, the global Peptide Therapeutics market will reach over $25 billion (Global Peptide Therapeutics Market, 2016). This dramatic market increase is driven by both growing incidences of cardiovascular and metabolic diseases, and also technological enhancements in peptide synthesis that include high-throughput approaches.

## Peptidomimetic-Based Therapy in Cardiovascular Disease

Attempts to prevent cardiovascular diseases usually involve control and improvement of causative risk factors such as hypercholesterolemia, inflammation, hyperglycaemia, obesity, insulin resistance, or high blood pressure. Limitations in currently available device therapies and pharmacologic drugs in CVD has prompted wider investigation into new treatment modalities such peptides and their mimetics.

## Apolipoprotein Mimetic Peptides

Dyslipidemia is one of most relevant risk factors for coronary artery disease. Therefore, one of the key goals of cardiovascular therapies is to reduce LDL cholesterol accumulation in the subendothelial space lining the artery wall, thereby preventing the progression of atherosclerosis and reducing the risk of heart attack and stroke. A plasma LDL cholesterol reduction of 1 mmol/L has been reported to reduce the risk of cardiovascular events by approximately 20% (Stoekenbroek et al., 2015). HDL is considered to promote the removal of free cholesterol from peripheral tissue and its transport to the liver for eventual clearance. ApoA-I, the major protein component of the HDL particle, is predominantly responsible for the anti-atherogenic properties attributed to HDL (Fisher et al., 2012). ApoA-I is critical for the process of reverse cholesterol transport and cellular cholesterol homeostasis. Several murine pre-clinical models of atherosclerosis have shown potent protective effects of apoA-I following prophylactic and therapeutic intervention (Gordon et al., 2011). Furthermore, genetic ablation of apoA-I in LDL receptor knockout mice, was shown to significantly promote atherosclerosis progression (Moore et al., 2003).

In addition to its role in cholesterol transport, other vascular beneficial effects have been attributed to apoA-I (Mangaraj et al., 2016). Recent studies have reported a potential anti-inflammatory role for apoA-I in the regulation of monocyte/macrophage recruitment to local sites of inflammation, via modulation of lipid rafts in cellular membranes which resulted in suppression of PI3K/Akt signaling (Iqbal et al., 2016). It also displays anti-oxidant properties as shown by its ability to inhibit LDL oxidation, remove lipid hydroperoxides, and also protect endothelial cells from apoptosis (Suc et al., 1997; Podrez, 2010; Rosenbaum et al., 2015). Furthermore, its structural homology with PSF has contributed to its anti-clotting and anti-aggregation effects on platelets which has strengthened its cardioprotective role (Yui et al., 1988). All in all, apoA-I is widely considered as a promising target for CVD treatment, and different therapeutic approaches have been developed to mimic its function.

ApoA-I is a 243 amino acid molecule with a secondary structure of 10 amphipathic αα-helices necessary for its interaction with lipids (Davidson et al., 1996). This secondary structure has been used as a template to design a range of apoA-I mimetic peptides. Although they are functionally similar to the native protein, they have unique structural properties (**Table 2**) (Stoekenbroek et al., 2015).

**Table 2:** Apolipoprotein mimetic peptides.

ApoA-1 peptide	Structure/Sequence	Clinical implications	Reference
18A	DWLKAFYDKVAEKLKEAF	First and shortest peptide reported to clear phospholipid	Venkatachalapathi et al., 1993
4F	Ac-DWFKAFYDKVAEKFKEAF-NH_2_	Anti-inflammatory, anti-oxidant and atheroprotective effects in experimental models	Li et al., 2004; Bloedon et al., 2008; Van Lenten et al., 2008
6F	DWLKAFYDKFFEKFKEFF	Potent anti-inflammatory, anti-oxidant and atheroprotective effects in mice; not require end blocking to be effective	Chattopadhyay et al., 2013; Navab et al., 2013
37pA	18A-P-18A	Cellular cholesterol eﬄux via ABCA1	Remaley et al., 2003
5A	18A-P-DWAKAAYDKAAEKAKEAA	Atheroprotective, anti-inflammatory, anti-oxidant. Specific for ABCA1 in cholesterol transport.	Amar et al., 2010; Tabet et al., 2010
ETC-642	PVLDLFRELLNELLEALKQKLK	Potent induction of cholesterol transport and increase of HDL fraction; anti-inflammatory, anti-atherosclerotic	Di Bartolo et al., 2011b; Iwata et al., 2011
FAMP	H-ALEHLFTLYEKALKALEDLLKKLL-OH	Enhance HDL biological function via ABCA1; atheroprotective	Uehara et al., 2013
**ApoE Peptide**			
ATI-5261	EVRSKLEEWFAAFREFAEEFLARLKS	Induction of ABCA1-mediated cholesterol transport; reduction of aortic lesion area and plaque lipid content	Bielicki et al., 2010
Ac-hE18A-NH_2_	Ac-LRKLRKRLLR-18A-NH_2_	Potent reduction in plasma cholesterol; clearing of atherogenic lipoproteins, reduction of atheroma plaque and improvement of endothelial function	Gupta et al., 2005; Datta et al., 2010

*Name, sequence and clinical implications of the most studied Apo mimetic peptides in different laboratories. Modified from (White et al., 2014). Other references used: (Di Bartolo et al., 2011b; Uehara et al., 2013).*

The first apoA-I mimetic peptide, 18A, was synthesized by Anantharamaiah et al. (1985; Venkatachalapathi et al., 1993). Subsequently, this 18 amino acid peptide has undergone numerous modifications to generate variant mimetic peptides with increased homology to apoA-I, higher lipid affinity and enhanced anti-atherogenic properties (Garber et al., 1992). An example of a such a variant is 4F, the most well-studied apoA-I mimetic, that reproduces the helical and amphipathic portion of apoA-I which is key for its function (Navab et al., 2005). Other peptides such as D-4F and L-4F, consist of the D- and L-isomers of the amino acids and show similar functionality as apoA-I, with D-4F being more stable via oral administration (Navab et al., 2002). However, although initially demonstrating potent anti-inflammatory, anti-oxidant, and atheroprotective effects in pre-clinical experimental models in apoE null mice and in human aortic cell cultures, 4F peptides have failed to show any efficacy in human trials (Li et al., 2004; Bloedon et al., 2008; Van Lenten et al., 2008; Watson et al., 2011).

Several other apoA-I mimetics have been developed to overcome some of the weaknesses of previous peptides. For example, the 6F peptide emerged as a promising apoA-I mimetic which did not require end blocking to be effective, and therefore reduced overall costs for synthesis. This peptide was also shown to possess potent anti-inflammatory, anti-oxidant, and atheroprotective effects in pre-clinical experimental models in LDL receptor-null mice (Chattopadhyay et al., 2013; Navab et al., 2013). 5A peptide was synthesized based on an existing 37 pA peptide structure to which five amino acids where replaced in order to decrease its cytotoxicity associated with its elevated lipid affinity (Remaley et al., 2003). In this way, 5A was less toxic and more specific to the ATP-binding cassette transporter A1(ABCA1) in cholesterol transport. Moreover, this apoA-I mimetic reduced pro-inflammatory adhesion molecule expression, neutrophil infiltration, and oxidative stress in animal models of inflammation in rabbits and also *in vitro* in human coronary artery endothelial cells (Tabet et al., 2010). 5A was also shown to be atheroprotective in pre-clinical mouse models and there are current proposals under consideration to take this mimetic forward into clinical trials (Amar et al., 2010).

ETC-642 is a 22 amino acid apoA-I mimetic peptide that offers numerous beneficial effects on LDL and HDL particles, including reduction of pro-inflammatory oxidized LDLs, potent induction of cholesterol transport, and increase of cholesterol content in the HDL fraction. It has also been attributed with significant anti-inflammatory properties in several studies of acute and chronic inflammation in rabbits, where it was shown to reduce TNFα induced expression of NF-Kb and endothelial adhesion molecule expression (Di Bartolo et al., 2011a,b). Furthermore, ETC-642 was shown to inhibit plaque formation in an experimental model of atherosclerosis in hyperlipidemic rabbits (Iwata et al., 2011).

In 2010, a systematic study of 22 different apoA-I mimetic peptides reported by D’Souza et al. (2010) showed that the structural modifications of each peptide were related with their different capacity and specificity of cholesterol eﬄux and their inhibitory effects on inflammation and LDL oxidation. In this analysis none of the peptides tested were found to be equally effective in all anti-atherogenic functions (D’Souza et al., 2010).

Many of these apoA-I mimetic peptides are in pre-clinical stages of development (Smith, 2010; White et al., 2014; Uehara et al., 2015). A newly described apoA-I mimetic peptide, called FAMP (Fukuoka University APOA-I mimetic peptide), has been reported to function via ABCA1 in a highly specific manner. This novel mimetic peptide has been shown to effectively enhance HDL biological function and it also has atheroprotective functions in apoE-deficient mice (Uehara et al., 2013).

More recently, apoE mimetic peptides were shown to have a beneficial impact on HDL functionality. ApoE is a 299 amino acid protein that plays an important role in clearing apoB-containing remnant particles mainly chylomicrons (that absorb lipids from the diet in the intestine), very low-density lipoproteins (VLDL, that transport triglycerides to tissues), and other lipoproteins that can be atherogenic (Bocksch et al., 2001). ApoE clears lipoproteins by LDL receptor-independent mechanisms. It also plays a crucial role in the regulation of plasma cholesterol levels, given that it contains an LDL binding domain in its structure (Hatters et al., 2006; Mahley et al., 2006). In addition, other beneficial effects have been attributed to apoE including anti-inflammatory, anti-oxidant, and anti-coagulant properties (Ali et al., 2005; Pham et al., 2005; Gaudreault et al., 2012).

Several mimetic peptides based on apoE structure have been recently designed (**Table 2**). Among them, ATI-5261 is a 36 amino acid peptide that has been reported to induce ABCA1-mediated cholesterol transport and reduce aortic lesion area and plaque lipid content in several pre-clinical models of atherosclerosis in mice (Bielicki et al., 2010). Anantharamaiah et al. (1985) developed various synthetic dual-domain apolipoprotein peptides which are structurally and functionally similar to apoA-I and apoE but mimic the cholesterol-lowering properties of apoE (Datta et al., 2001; Sharifov et al., 2011). The most characterized is Ac-hE18A-NH_2_, composed of a region of the LDL binding domain of apoE linked to the apoA-I mimetic 18A (Sharifov et al., 2011). This peptide was shown to dramatically reduce plasma cholesterol in several dyslipidemic animal models and had the extra advantage of clearing atherogenic lipoproteins due to the presence of the LDL binding domain, resulting in the reduction of atheroma plaque formation and the improvement of endothelial function (Gupta et al., 2005; Datta et al., 2010). This novel intravenously administered-peptide has been assigned orphan drug status and, under the name AEM-28, is currently undergoing initial (phases 1 and 2) clinical assessment (White et al., 2014).

Many of these peptides are still in pre-clinical phases of development and to date it has been difficult to identify an efficacy parameter for apo mimetics in human trials collectively. One major reason for the discrepancy observed in humans and mice could be differences in the composition of lipid associated proteins. A study from Gordon et al. (2015) utilized a mass spectrometry approach to demonstrate a high degree of shared homology amongst a range of proteins associated with LDL and HDL. However, a small minority of proteins did exhibit significant differences which could reflect in major metabolic differences between species (Gordon et al., 2015).

Surprisingly, there are no reported studies which have compared the efficacy of statins versus apoA-I mimetics in humans to date. LDL-lowering statin therapy is currently considered the ‘gold standard’ treatment for CVD. Statins are very effective and safe in atherogenic dyslipidemia treatment. However, they have shown to lack benefit for retarding residual adverse cardiovascular events. Even under optimal statin treatment, patients with familial hypercholesterolemia present with high level of LDL cholesterol and there are also patients who are intolerant or unresponsive to statins, highlighting a potential role for the use of apo mimetics in such patients (Boekholdt et al., 2013; Ahn and Choi, 2015; Uehara et al., 2015). Given the current interest in this field we can expect to have novel apo mimetic peptides in the near future to aid in the prevention and treatment of patients with cardiovascular disorders.

## SOCS1-Derived Mimetic Peptides

It is widely accepted that inflammation participates in all stages of atherosclerosis, from its initiation to its thrombotic complications (Libby et al., 2011). Therefore, targeting inflammatory mediators that dynamically take part in chronic inflammation which underlies disease could be an interesting clinical strategy. In this context, SOCS proteins, which are at the crossroad of multiple inflammatory pathways, have recently emerged as a potential therapeutic target with anti-inflammatory functions (Linossi et al., 2013; Trengove and Ward, 2013). SOCS are negative-feedback regulators of the JAK/STAT pathway, which drive the production of cytokines and inflammatory factors that affect atherosclerotic processes, including leukocyte recruitment, migration, and proliferation of vascular cells, foam cell formation and apoptosis (Marrero, 2005; Miklossy et al., 2013). Among the eight members of this family of proteins (SOCS1-7 and CIS), SOCS1 and SOCS3 are of particular interest because they contain a conserved 12-residue KIR that is involved in direct suppression of JAK activity and they have also been linked to a variety of pro-inflammatory and pro-atherogenic factors including lipoproteins, lipids, high glucose, angiotensin II, and insulin (Alexander, 2002; Yoshimura et al., 2007; Liang et al., 2013). Furthermore, experimental studies in mice and murine aortic cells demonstrate that SOCS overexpression reduces inflammation and cardiovascular disease (Tajiri et al., 2012; Qin et al., 2014). Studies based on peptides mimicking the action of SOCS proteins have been reported in different experimental settings (**Table 3**). The first SOCS mimetic peptide developed was JAK2 Tkip (Flowers et al., 2004). This short 12-mer peptide was shown to suppress the expression of inflammatory cytokines such as TNFα, inhibit lymphocyte proliferation as well as IFNγ-induced macrophage activation and NO production in mice (Mujtaba et al., 2005; Ahmed et al., 2009). SOCS1-KIR peptidomimetic was reported to inhibit STAT activation by Th1 and Th17 cytokines in leukocytes as well as suppress the expression of pro-inflammatory mediators and activation and migration of vascular cells and macrophages *in vitro* (Ahmed et al., 2015). SOCS1-KIR was also shown to be atheroprotective in a type I diabetes mouse model, decreasing vascular plaque accumulation of lipids, macrophages, and T cells, and reducing aorta expression of pro-inflammatory cytokines and chemokines (Recio et al., 2014). More recently, this SOCS1-KIR peptide was demonstrated to further improve diabetes associated-renal damage in mice as well as reduce inflammation and fibrosis in diabetic kidneys (Recio et al., 2016).

**Table 3:** SOCS mimetic peptides.

Peptide	Structure/Sequence	Properties	Reference
Tkip	WLVFFVIFYFFR	Anti-inflammatory	Mujtaba et al., 2005; Ahmed et al., 2009
S0CS1-KJR	DTHFRTFRSHSDYRRI	Atheroprotective, anti-inflammatory	Recio et al., 2014, 2016; Ahmed et al., 2015
NewSOCSl-K1R	DTHFRTFRSH	Anti-inflammatory	Doti et al., 2012
PS-5	DTC(Acm)RQTFRSH	Anti-inflammatory	Doti et al., 2012; Madonna et al., 2013

*Name, sequence, and properties of the most relevant SOCS mimetic peptides in different laboratories. Modified from (Doti et al., 2012; Ahmed et al., 2015).*

Doti et al. (2012) recent studies focused on identifying new improved mimetic peptides of the KIR region of SOCS1 but with enhanced affinity, stability, and potency profiles. Among them, PS-5 was highlighted because it bound to JAK2 more efficiently than KIR and also prevented the IFNγ-induced activation of STAT and its downstream inflammatory effects (Doti et al., 2012; Madonna et al., 2013).

In summary these peptidomimetics emerge not only as potent anti-inflammatory agents but also as promising future drugs in the treatment of cardiovascular complications in diabetic patients.

## Incretin Mimetics

In diabetic patients, the control of blood glucose levels is a major goal to prevent further tissue damage and cardiovascular events such as stroke, heart attack, or end-stage renal disease (Snell-Bergeon and Wadwa, 2012). Incretin mimetic-based therapies, with particular focus on GLP-1R agonists and DPP4 inhibitors, are currently leading therapeutic agents available for type 2 diabetes treatment (Drucker and Nauck, 2006). As peptidomimetics, GLP-1R agonists mimic the actions of the endogenous hormone GLP-1 in that they stimulate glucose-induced insulin secretion, suppress glucagon secretion and hepatic glucose production and delay gastric emptying. In addition, GLP-1 has been reported to enhance peripheral glucose disposal (very important in diabetes) as well as promote pancreatic beta cell growth and differentiation (Drucker and Nauck, 2006; Meier, 2012). Furthermore, GLP-1R agonists can act both in a short and long-term manner, allowing personalized patient regimes to be offered (Neumiller, 2015).

DPP-4 is the enzyme that inactivates GLP-1, therefore its inhibition emerges as another potential target to increase circulating levels of GLP-1 thereby increase circulating incretin levels (Deacon et al., 1998, Deacon, 2011).

Incretin mimetics present other favorable properties such as a low hypoglycaemia risk, the ability to address postprandial hyperglycemia (DPP-4 inhibitors and short-acting GLP-1R agonists), and potential for weight reduction (GLP-1R agonists; Neumiller, 2015).

Interestingly, besides regulation of glucose homeostasis, GLP-1 mimetic peptides have also been shown to exert cardioprotective effects in cardiovascular-related death, non-fatal MI, and non-fatal stroke (Advani et al., 2013; Wroge and Williams, 2016).

In the last decade, three different GLP-1R agonists have been approved for clinical use; Exenatide, first approved in 2005, Liraglutide and Lixisenatide; Albiglutide, Dulaglutide, and Semaglutide are in last phases of evaluation (**Table 4**) (Eng et al., 1992; Meier, 2012). One particular feature of Exenatide and Lixisenatide is that, in contrast to the endogenous GLP-1 which is degraded within 1–2 min by DDP-4, they are both DDP-4-resistant. While Exenatide requires a twice daily dosing regime, Lixisenatide can be given once a day because it has a higher affinity for GLP-1R. However, they have a similar half-life (2–4 h; Madsbad et al., 2011; Bhavsar et al., 2013; Kalra et al., 2016). Extended stability and longer half-life of these compounds would be favorable. In contrast, Liraglutide, can be administered once a day and has half-life of 13 h as a result of a modification to the peptide backbone with palmitic acid. This compound has been reported to induce significant weight loss and reduce blood pressure as well as diabetes prevalence in type 2 diabetic patients (Juhl et al., 2002; Russell, 2013).

**Table 4:** Incretin mimetic peptides.

Peptide	Structure/Sequence	Dosing	Status	Reference
Exenatide	39 aa peptidase-resistant peptide	s.c. twice daily or once weekly	Approved for T2 diabetes	Madsbad et al., 2011; Bhavsar et al., 2013
Liraglutide	31 aa peptide linked to lipid	s.c. once daily	Approved for T2 diabetes	Juhl et al., 2002; Russell, 2013
Lixisenatide	44 aa peptidase-resistant peptide	s.c. once daily	Approved for T2 diabetes	Kalra et al., 2016
Albiglutide	Tandem repeat of 30 aa peptide fused with human albumin	s.c. once weekly	Regulatory review-	Rosenstock et al., 2009
Dulaglutide	46 aa peptide fused with IgG4 Fc	s.c. once weekly	Phase III for T2 diabetes	Jimenez-Solem et al., 2010
Semaglutide	37 aa acylated peptide	s.c. once weekly	Phase III for T2 diabetes	Nauck et al., 2016
HM11260C, LAPS-Exendin	Exendin-4 analog conjugated to human Ig fragment	s.c. once weekly or once monthly	Phase II for T2 diabetes	Kaspar and Reichert, 2013
NN9926, OG9S7GT	GLP-1 analog; long-acting	Oral	Phase I for T2 diabetes	Kaspar and Reichert, 2013; Mittermayer et al., 2015
ZY0G1	GLP-1 agonist	Oral	Phase I for T2 diabetes	Kaspar and Reichert, 2013; Mittermayer et al., 2015
TT401	Dual agonist	s.c. once weekly	Phase I for T2 diabetes, obesity	Kaspar and Reichert, 2013; Mittermayer et al., 2015; (“TransitionTherapeutics announces results of clinical study of type 2 diabetes drug candidate TT-401,” 2013)

*Name, structure, administration dose and clinical status of the most relevant incretin mimetic peptides. Modified from (Irwin and Flatt, 2015).*

Albiglutide and Dulaglutide are incretin mimetics with increased half-life (4–7 days) which allow for weekly administration. This extension of their half-life is feasible owing to their fusion with different molecules that confer them stability (i.e., human albumin; Rosenstock et al., 2009). There are two completed studies with Albiglutide that demonstrate the safety and efficacy of weekly, subcutaneously injected doses compared to other treatments such as Liraglutide or insulin. Dulaglutide is administered by subcutaneous injection once weekly for up to 24 months at seven doses (Jimenez-Solem et al., 2010). Semaglutide is to date the last one in the list of GLP-1 mimetics which are under clinical assessment. Phase III studies of this compound confirm that it can be administered subcutaneously once weekly and it improves glycaemic control in type 2 diabetes patients in a superior way than Exenatide. Semaglutide also reduces the risk of major cardiovascular events and decreases appetite and food intake, therefore becoming an interesting drug to be used in obese patients (Nauck et al., 2016).

Incretin mimetics are the current preferred drug to treat type 2 diabetes owing to their wide range of beneficial effects. However, although many of them are already in clinical use, evolution of this group of peptides is not complete. There are still a high number of studies focused on improving patient convenience and compliance so looking for strategies to reduce dosing frequency or developing oral administrated compounds.

## Annexin-A1 Mimetic Peptides

Given that MI remains a major cause of death worldwide and the current therapies based in revascularization of the ischemic tissue (anti-oxidants and calcium channel blockers) have shown insufficient success, novel strategies are needed to treat patients with MI. In this context, the therapeutic potential of glucocorticoid-regulated anti-inflammatory mediator annexin-A1 has been demonstrated in different systemic inflammatory disorders. Annexin-A1 is a glucocorticoid-inducible 37 kDa protein, highly expressed by macrophages, that activates the family of formyl peptide receptors and inhibits different processes related to myocardial reperfusion injury such as polymorphonuclear leukocyte activation, migration, and infiltration (Ambrose et al., 1992; De Caterina et al., 1993; La et al., 2001; Perretti and Gavins, 2003; Qin et al., 2015).

Due to the potent anti-inflammatory and cardioprotective properties of endogenous annexin-A1, several studies utilized experimental models to examine the role of the exogenous protein and its derived peptides (Perretti and Gavins, 2003). The main benefits attributed to annexin-A1 peptide mimetics include cardioprotection based on their anti-inflammatory effect to preserve myocardial viability after MI but also other inflammation-independent properties that directly protect cardiomyocytes viability and contractile function (Qin et al., 2015). The subcutaneous administration of annexin-A1 N-terminal derived peptide Ac2-26 has been shown to confer protection against ischemia-reperfusion injury by reducing myeloperoxidase activity and IL-1β levels in the infarcted heart, as well as down-regulate monocyte accumulation and inhibit phagocytic activity of macrophages in different rodent experimental models (Getting et al., 1997; La et al., 2001). Another annexin-A1 mimetic is CGEN-855A, a 21 amino acid peptide displays anti-inflammatory effects by inhibition of polymorphonuclear neutrophils recruitment and also provides protection against ischemia-reperfusion-mediated injury to the myocardium after being injected intravenously in mice (Hecht et al., 2009).

## Conclusion and Future Perspectives

The view that peptides hold multiple properties as therapeutics, including suitable pharmacokinetic profiles, low toxicity and immunogenicity, and desirable solubility features, is broadly accepted. Since many of the classical limitations they possess to act as drug agents are being overcome by improving techniques and modifications, the use of peptides and peptidomimetics as a therapeutic strategy is growing.

Although the use of these molecules in CVD treatment is gaining traction, more effort is needed to improve therapeutic potential. With further studies of the structures, interactions, and functions of proteins and mediators implicated in CVD, more peptides will be discovered and developed. With this strategy, the use of these molecules could provide good opportunities for cardiovascular prevention and treatment, surpassing some of the limitations of current therapies.

## Author Contributions

CR and FM drafted the manuscript; AI drafted part of the manuscript and designed it; NM and VDF drafted part of the manuscript and revised it critically for intellectual content.

## Conflict of Interest Statement

The authors declare that the research was conducted in the absence of any commercial or financial relationships that could be construed as a potential conflict of interest.
